# 82 Does Assessing Frailty Make a Difference?

**DOI:** 10.1093/jbcr/iraf019.082

**Published:** 2025-04-01

**Authors:** Karen Richey, Derek Murray, Hunter Cooley, Tiffany Hockenberry, Philomene Spadafore, Kevin Foster

**Affiliations:** Diane & Bruce Halle Arizona Burn Center; Diane & Bruce Halle Arizona Burn Center; Valleywise Health and Arizona Burn Center; Diane & Bruce Halle Arizona Burn Center; Diane & Bruce Halle Arizona Burn Center; Diane & Bruce Halle Arizona Burn Center

## Abstract

**Introduction:**

As the Silver Tsunami hits healthcare, attention has been turned towards optimizing care for this population. The concept of frailty has proliferated, yet at the 2019 National Institute of Aging Frailty Symposium it was noted that while multiple instruments had been developed to measure frailty, implementation of assessment was slow. They also noted a lack of evidence that doing so aided clinical decision making or improved outcomes. To date, frailty work within the burn population has been retrospectively performed. The purpose of this quality improvement (QI) project was to prospectively examine the utility of frailty scoring in a burn center.

**Methods:**

This prospective, observational QI project was conducted over the first 3 months following implementation of a frailty scoring system, the Clinical Frailty Scale (CFS), for patients age ≥ 60 years. Patients received a numeric frailty score and a dichotomous categorization of frailty. Patients with scores >3 were considered frail (F) and scores ≤ 3 as not frail (NF). Retrospectively the Modified Frailty Index (mFI-5) was calculated. Basic demographic and hospital data was collected, descriptive statistics were calculated. Student’s t-test was used to compare F to NF.

**Results:**

A total of 124 patients age ≥ 60 years, were admitted during the 3-month period. Frailty scoring was not completed on 7 due to death or discharge within 48 hours of admission. Thus 117 were evaluable. The mean age was 72 years (± 7.842, range 60-92), the majority (85%) had sustained a burn injury. For those with burns the mean TBSA was 4.3% (±1.68, range 8.6-13.7%). Mean CFS was 4.34 (±1.68, range 1-8) with 82 (70%) deemed frail. Mean ventilator days 2.69, ICU days 3.573, and length of stay 13 days. The average number of comorbidities was 3.58/patient and 11 (9%) died. Of those who lived only 24 (22%) were able to return to their prior living situation at discharge. When comparing F (n=82) to NF (n=35), significant differences were found in their CFS score, F 5.18 vs NF 2.37 (p <.0001), MFI-5 score F 1.87 vs NF 0.97 (p <.0001) and return to prior living situation F 21% vs NF 44% (p <.01). There was no significant difference in age, %TBSA ventilator/ICU/LOS days or mortality.

**Conclusions:**

To our knowledge this is the first report of frailty scoring performed prospectively in a burn center population. Based on our findings the immediately recognized value for frailty scoring may be in the discharge planning process. Utilizing the pre-injury score may also prove useful in an outpatient rehabilitation setting to determine if patients have returned to their pre-injury state or have functionally declined and require additional therapy. The impact of frailty scoring on decision making and the resultant outcomes remains unknown.

**Applicability of Research to Practice:**

A multi-center, prospective, observational trial should be conducted to fully determine the utility of frailty scoring to optimize care in a burn population.

**Funding for the Study:**

N/A

